# A Viscoelastic Modeling for Failure Analysis of Human Vertebral Bone Undergoing Quasi-Static and Dynamic Compression

**DOI:** 10.3390/bioengineering13070747

**Published:** 2026-06-26

**Authors:** Mahmood Allahyari, Mehran Fereydoonpour, Asghar Rezaei, Ghodrat Karami

**Affiliations:** 1 Department of Mechanical Engineering, North Dakota State University, Fargo, ND 58102, USA; mahmood.allahyari@ndsu.edu (M.A.); mehran.fereydoonpour@ndsu.edu (M.F.); 2 Department of Mechanical Engineering, Dariun Branch, Islamic Azad University, Dariun 7146704949, Iran; 3 Department of Physiology and Biomedical Engineering, Mayo Clinic, Rochester, MN 55905, USA; rezaei.asghar@mayo.edu

**Keywords:** vertebral biomechanics, trabecular bone, viscoelasticity, strain-rate dependence, bone density

## Abstract

Vertebral fractures are among the most common skeletal injuries and present significant clinical and biomechanical challenges, particularly in older adults and individuals with low bone density. Accurate prediction of vertebral mechanical response and failure under varying loading conditions is essential for improving understanding of spinal injury mechanisms. This study develops a density-dependent viscoelastic analytical model to predict the stiffness and fracture force of human vertebral specimens subjected to different compression rates. The vertebral body is represented as a composite structure consisting of a cortical shell and a trabecular core. Cortical bone is modeled as a linear elastic material, whereas trabecular bone is described using a Kelvin–Voigt viscoelastic formulation. Density-dependent constitutive relationships are incorporated for the elastic modulus and viscous coefficient of trabecular bone. Unknown material parameters are identified through optimization using the Nelder–Mead algorithm, based on experimental compression data from cadaveric vertebral specimens tested under quasi-static and dynamic loading conditions. The calibrated model reproduced the overall trend of specimen-to-specimen mechanical variation observed experimentally. Predicted stiffness values were in reasonable agreement with measured data. Fracture force predictions showed moderate agreement for dynamically tested specimens (R^2^ = 0.60), which improved to R^2^ = 0.88 after exclusion of one statistically identified outlier. Compared with a purely linear elastic formulation, the proposed viscoelastic model demonstrated modest improvement in stiffness prediction and more substantial improvement in fracture force prediction. These findings indicate that incorporating density-dependent viscoelastic effects improves representation of vertebral mechanical behavior, particularly at higher loading rates. Owing to its simplicity and computational efficiency, the proposed model requires only limited imaging input and may be useful for future biomechanical investigations, rapid screening, and injury risk prediction.

## 1. Introduction

Vertebral fractures are one of the most common bone injuries and are a major clinical problem, especially in older people and in patients with low bone density [1,2,3]. These fractures can lead to severe pain, changes in the shape of the spine, reduced mobility, and a greater risk of future fractures [2,3]. As a result, predicting the mechanical behavior and failure of vertebrae has become an important subject in spine biomechanics and injury research.

The mechanical behavior of trabecular bone and vertebral structures has been investigated using different experimental, analytical, and computational methods. Multiscale micromechanical models have been proposed to describe the anisotropic mechanical properties of vertebral trabecular bone by Haj-Ali et al. [4]. Green et al. [5] have used experimental studies, together with finite element analysis to examine stress–strain behavior and the start of damage in trabecular bone. In addition, models such as poroelastic formulations have been developed to study the effect of fluid flow inside the porous trabecular structure by Lim and Hong [6].

Finite element models have been widely used to investigate the mechanical behavior of spinal components and vertebrae under various loading conditions. In addition, viscoelastic formulations have been applied to examine load sharing within spinal structures at different loading rates [7] and to identify critical loading conditions through nonlinear analyses [8]. Experimental studies have also characterized the viscoelastic behavior of trabecular bone across multiple length scales using techniques such as dynamic mechanical testing and nanoindentation [9]. Many studies have demonstrated that the mechanical properties of trabecular bone are strongly dependent on apparent density. Several works have reported power-law relationships between elastic modulus and density, although these relationships vary with anatomical location and microstructural characteristics [10,11,12,13]. Wu et al. [14] have also shown that these properties can vary significantly and that density plays an important role in bone stiffness and strength. While density-dependent elastic behavior has been extensively studied, relatively few analytical models incorporate both density dependence and strain-rate sensitivity within a unified framework. In addition to density effects, trabecular bone exhibits pronounced strain-rate dependence. Under dynamic loading conditions such as falls, sports impacts, or motor vehicle accidents, its mechanical response can differ significantly from that observed under quasi-static loading. Previous research has shown that both stiffness and strength increase with increasing strain rate [11,12,13]. This behavior is primarily attributed to the porous microstructure of trabecular bone, which contains marrow and fluid and exhibits time-dependent mechanical effects [15]. Several studies have investigated the viscoelastic properties of trabecular bone using creep and stress relaxation tests. For example, Manda et al. [16] examined the relationship between viscoelastic behavior and bone volume fraction using creep tests on bovine trabecular bone, and later extended this work to include nonlinear viscoelastic effects [17]. However, most of these experiments were performed at low strain rates (about 0.01 s^−1^), which may not fully represent traumatic conditions where strain rates are much higher. As a result, viscoelastic parameters obtained from low strain-rate testing may not completely describe the behavior of vertebral bone under dynamic loading conditions. Including strain-rate effects by using compression tests at different loading rates may improve prediction accuracy. Although many studies have investigated density-dependent elastic properties, only a limited number of studies have included density-dependent viscoelastic parameters in analytical models of vertebral mechanics. Developing models that consider both density changes and rate-dependent behavior is still an important challenge.

The human spine is routinely exposed to complex, multi-axial biomechanical demanding environments involving bending, torsion, tension, and compression. Among these loading modes, axial compression represents the primary physiological mechanism responsible for clinical spinal failure, specifically Vertebral Compression Fractures (VCFs). VCFs constitute a critical global healthcare burden, highly prevalent in elderly populations and individuals suffering from metabolic bone disorders such as osteoporosis. Under physiological conditions, the anterior column of the vertebra bears the vast majority of axial compressive loads during daily activities. Consequently, characterizing the dynamic, time-dependent response and viscoelastic failure of the vertebral body under pure compression is a fundamental prerequisite to understanding the biomechanical onset of these failures.

This study presents an analytical model to predict the mechanical response and fracture force of human vertebral specimens under different loading rates. It also investigates the effect of including viscoelastic behavior compared with a purely linear elastic model. The model incorporates density-dependent elastic properties together with a Kelvin–Voigt representation of trabecular bone. Unlike many previous studies in which viscoelastic parameters were derived primarily from low strain-rate creep or relaxation experiments, the present approach identifies these parameters directly from vertebral compression tests performed at different loading rates.

In this context, although finite element methods can provide detailed predictions of vertebral mechanics, they usually require complex image processing, specialized expertise, and high computational cost. Therefore, the analytical model presented in this study can provide a practical and computationally efficient approach for vertebral evaluation.

## 2. Materials and Methods

### 2.1. Vertebral Specimens and Experimental Data

Experimental data used in this study were obtained from a previously published biomechanical investigation on cadaveric spinal segments conducted by Rezaei et al. [18] at the Mayo Clinic. In the original study, 28 spinal specimens were tested under compression loading conditions. From these specimens, 10 intact vertebrae originating from the thoracic and lumbar regions (spanning T6, T7, T8, T9, T12, L1, and L3 anatomical levels) were selected for the present study. As noted in the specimen nomenclature, the last three alphanumeric characters of each Bone ID directly represent its precise anatomical level. Three vertebrae representing these anatomical levels were tested under quasi-static loading, and seven were tested at higher strain rates to simulate fracture conditions. The tests gave force–displacement curves, which were used to calculate stiffness and fracture force for each specimen. These results were used as reference data to calibrate the model in this study.

To derive the physical bone density from quantitative computed tomography (QCT) data, the raw QCT scans were processed using Mimics medical imaging software Ver. 22.0 (Materialise, Ann Arbor, MI, USA) to segment the vertebral geometry. The continuous gray-scale voxel intensities covering the bone domain were partitioned into discrete material bins within the software. The average Hounsfield Unit (HU) values were then linearly transformed into physical ash density ($\rho_{ash}$) via a scan-specific calibration protocol using a reference phantom (Mindways Inc., Austin, TX, USA). The transformation followed a calibrated relationship of the form: $\rho_{ash}=m\cdot HU+n$, where the calibration constants m and n were uniquely calculated for each scan from the reference rods of known density, consistent with validated QCT density-mapping protocols [18].

The constitutive formulation of the developed viscoelastic framework is intrinsically density-dependent, utilizing the continuous ash density extracted from the QCT as a primary governing parameter. The underlying human cadaveric dataset was characterized by a diverse clinical spectrum of bone qualities, encompassing healthy, osteopenic, and severely osteoporotic vertebral bodies. This variation in bone status across the specimen cohort ensured that the predictive capacity and mathematical stability of the global optimization framework were rigorously evaluated under both robust physiological baselines and advanced metabolic bone degradation states.

**Figure 1:**
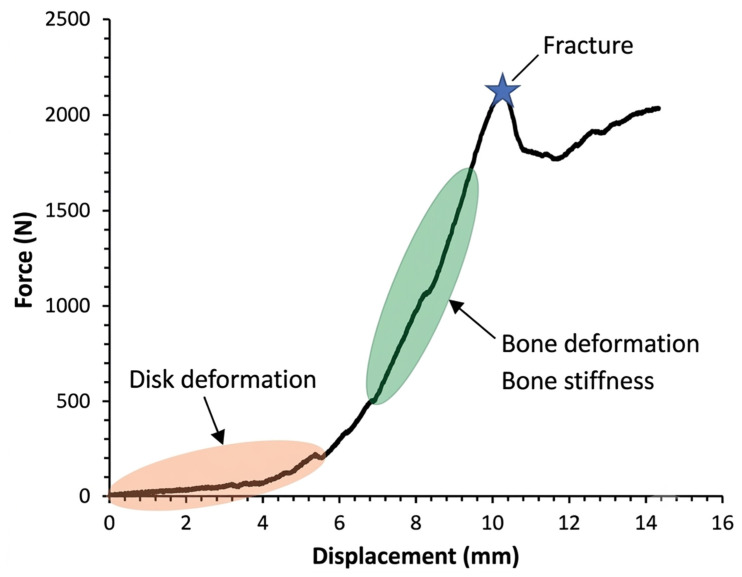
Schematic representation of the experimental force–displacement curve demonstrating the decoupling procedure of the intervertebral disc deformation (nonlinear toe region) from the linear response and subsequent macro-structural fracture phase of the isolated vertebral body.

During the underlying experimental tests, the presence of multi-level spinal motion segments meant that the initial non-linear phase of the experimental force–displacement curve (the characteristic ‘toe region’) was heavily dominated by the compliance, settling, and loading of the adjacent intervertebral discs (Figure 1). To eliminate this confounding structural effect and isolate the true bone response, the experimental stiffness used in the global Nelder–Mead optimization function was calculated exclusively from the subsequent, highly linear domain. In this linear phase, the adjacent discs are already fully compressed and act as a rigid, fluid-filled buffer (as schematically illustrated in Figure 1). This ensures that any incremental load and displacement are directly translated into the intrinsic elastic and viscoelastic deformation of the isolated vertebral body composite until the ultimate peak fracture force is reached.

### 2.2. Image-Based Density and Geometric Measurements

QCT images were used to obtain geometric and density-related parameters for each vertebra. For each specimen, the vertebral body was divided into cortical and trabecular regions using several axial CT slices. The density and cross-sectional area of the cortical and trabecular regions were measured in each slice. These values were averaged along the vertebral height to obtain the mean cortical density, trabecular density, cortical cross-sectional area, and trabecular cross-sectional area for each specimen. The average values were used as input parameters in the analytical model. The specimen specifications are shown in Table 1.

In Table 1, the last two or three characters of each Bone ID (e.g., T6, T12, L1, L3) indicate the precise anatomical level of the vertebral specimen within the thoracic or lumbar spine.

### 2.3. Model Description

**Figure 2:**
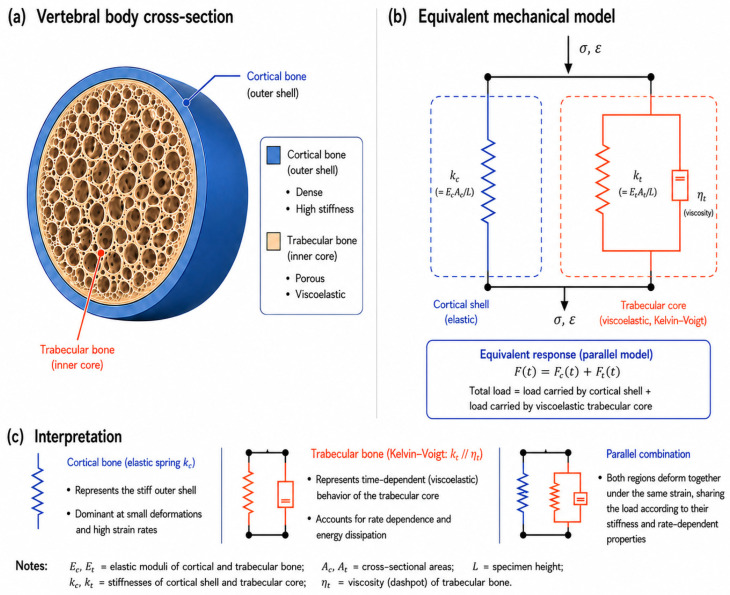
Illustrates the vertebral body cross-section (**a**), the equivalent mechanical model (**b**), and their physical interpretation (**c**).

The vertebra was represented using an analytical model in which the vertebral body was approximated as a cylindrical structure composed of two mechanically distinct components: cortical bone and trabecular bone. A schematic illustration of the vertebral body, including the cortical shell and trabecular core, together with the corresponding equivalent mechanical model, is presented in Figure 2. The cortical region was modeled as a linear elastic material, whereas the trabecular region was described using a viscoelastic formulation. Under axial compression, cortical and trabecular bone experience the same overall deformation. Therefore, the two components were assumed to act mechanically in parallel, resulting in identical strains in both trabecular and cortical bone, i.e., $\varepsilon =\varepsilon_{t}=\varepsilon_{c}$. where ε denotes the overall axial strain of the vertebra, and $\varepsilon_{t}$ and $\varepsilon_{c}$ are the trabecular and cortical bone strains, respectively. The viscoelastic behavior of the trabecular bone was represented using a Kelvin–Voigt model consisting of an elastic spring and a viscous dashpot connected in parallel. The Kelvin–Voigt formulation has been widely used to characterize the time-dependent mechanical response of biological tissues [19]. Accordingly, the constitutive relation for trabecular bone can be written as

(1) $$\sigma_{t}=E_{t}\varepsilon +ƞ\dot{\varepsilon }$$

where $\sigma_{t}$ is the stress in the trabecular part, $E_{t}$ is the trabecular elastic modulus, ƞ is the viscous coefficient, and $\dot{\varepsilon }$ denotes the strain rate.

The cortical component was assumed to behave as a purely elastic material, described by $\sigma_{c}=E_{c}\varepsilon$ Since the cortical and trabecular components act in parallel, the total stress carried by the vertebra can be expressed as the sum of the stresses in each phase,

(2) $$F=F_{c}+F_{t}$$

where $F_{c}$ and $F_{t}$ denote the forces carried by cortical and trabecular bone, respectively. These forces can be expressed as $F_{c}=$ $A_{c}$ $\sigma_{c}$ and $F_{t}=$ $A_{t}$ $\sigma_{t}$ where $A_{c}$ and $A_{t}$ represent the cross-sectional areas of the cortical shell and trabecular core. Substituting the constitutive relations into the force equilibrium equation yields

(3) $$F={A_{c}E}_{c}\varepsilon +A_{t}\left(E_{t}\varepsilon +ƞ\dot{\varepsilon }\right)$$

### 2.4. Density-Dependent Material Formulation

To consider differences in bone density between specimens, density-dependent material relationships were used. The elastic modulus of the bone was defined as a power-law function of density. This type of relationship has been widely reported in experimental and analytical studies on trabecular bone [20,21,22].

(4) $$E=a\rho^{b}$$

where E is the elastic modulus, ρ is the ash density, a and b are material constants obtained through parameter calibration. In order to account for density-dependent viscoelastic behavior, the Kelvin-Voigt model’s viscous coefficient was also defined as a linear function of bone density. The well-established relationship of trabecular bone mechanical characteristics on density shown in the literature [23,24,25,26,27] is consistent with this idea. The relationship is stated as follows,

(5) ƞ=cρ+d

where c and d are constants determined through the optimization procedure.

This formulation enables the analytical model to capture both the density dependence of bone stiffness and the strain-rate sensitivity associated with the viscoelastic behavior of trabecular bone.

### 2.5. Fracture Force Estimation

The compressive fracture force of each vertebral specimen is estimated using the effective modulus (*E_eff_*) predicted by the model. This modulus, which includes both elastic and viscous effects, was then used to estimate the corresponding stress response. The combined contribution of the cortical and trabecular regions was taken into account when calculating the effective modulus of the vertebral column. The effective modulus can be written as follows using the Kelvin–Voigt formulation:

$$E_{eff}=\frac{A_{c}E_{c}+A_{t}\left(E_{t}+ƞ\frac{\dot{\varepsilon }}{\varepsilon }\right)}{A_{total}}$$

where the cross-sectional areas of cortical bone, trabecular bone, and their total area are represented by $A_{c}$, $A_{t}$, and $A_{total}$, respectively. Based on this, the effective modulus and the shape of the specimen were used to calculate the stiffness of each specimen. The compressive stress was calculated by assuming linear elastic behavior up to failure $\sigma =E_{eff}\varepsilon$ where $E_{eff}$ represents the effective modulus derived from the analytical model. To estimate the fracture condition, a critical compressive strain of 10% was assumed for vertebral failure. This assumption has been widely adopted in previous experimental and computational studies of vertebral compression behavior [28,29,30]. The corresponding fracture force was then calculated as $F_{fracture}=\sigma_{f}A_{total}$. Where $\sigma_{f}$ is the stress at the assumed failure strain.

### 2.6. Normalization of Specimen Geometry

Because vertebral specimens differ in size and geometry, the experimental force–displacement data were normalized to remove geometric effects. The applied force was divided by the cross-sectional area of the vertebra, and the displacement was divided by the specimen height. This procedure yields a normalized stiffness that reflects intrinsic material behavior rather than geometric differences among specimens.

### 2.7. Parameter Calibration Identification

The experimental data used to calibrate the model were taken from a published study [18]. These data come from compression tests performed on human lumbar vertebral specimens under controlled loading conditions. The available dataset is somewhat sparse because of the inherent limits of experimental testing of biological tissues, such as restricted specimen availability, heterogeneity in bone quality, and testing complexity. However, the dataset reflects the general pattern of the mechanical response across the explored density range and offers a suitable basis for model calibration despite the small number of data points and inherent variability.

Optimization methods are widely used in structural and biomechanical engineering to identify parameters and improve design. For example, they have been used to find the best placement of piezoelectric patches to reduce stress in smart structures [31,32]. In the present study, an optimization procedure based on the Nelder–Mead algorithm was employed to identify the unknown material parameters of the proposed analytical model.

The unknown material constants a, b, c, and d, which define the density-dependent elastic and viscous relationships, were determined using the experimental data obtained from biomechanical testing. For each specimen, the analytical model was used to estimate the mechanical stiffness based on the specimen-specific geometric parameters and density measurements. The optimal parameters were obtained by minimizing the normalized sum of squared differences between the model predictions and the experimentally measured stiffness values across all specimens. The error function was defined as. ·∑i=1na,b,c,dminEeffmodel,i−knormexp,ik¯normexp2, where $E_{effmodel,i},k_{normexp,i}$ denote the predicted effective modulus and normalized experimentally measured stiffness values for specimen *i*, respectively, ${\bar{k}}_{normexp}$ represents the mean normalized experimental stiffness across all specimens, and *n* is the total number of specimens. The experimental stiffness was determined by calculating the slope of the force-displacement curve, whereas the projected stiffness was achieved using the effective modulus obtained from the viscoelastic model. The Nelder–Mead optimization algorithm was used to determine the parameter values that minimize this objective function.

The parameters a, b, c, and d, listed in Table 2 were obtained by fitting Equations (4) and (5) to the experimental data using a nonlinear least-squares optimization approach. Specifically, the parameters were determined by minimizing the normalized sum of squared differences between the model-predicted effective modulus and the experimentally measured normalized stiffness values across all specimens.

The objective function was defined based on the normalized error between $E_{effmodel}$ and $k_{normexp}$, where the experimental stiffness was calculated from the slope of the force–displacement curve. The optimization process was implemented in MATLAB R2023a (MathWorks Inc., Natick, MA, USA) using the Nelder–Mead algorithm [33].

### 2.8. Evaluation of Model Predictions

The predictive ability of the analytical model was evaluated by comparing the model results with the experimental measurements. First, relationships between density and both the elastic modulus and viscous coefficient were obtained using the calibrated parameters. These relationships were used to estimate the mechanical behavior of each specimen. The effective modulus and compressive fracture force of each vertebra were calculated using the predicted material properties and specimen geometry. The coefficient of determination (R^2^) was used to evaluate model performance, and scatter plots were used to visually compare the predicted and experimental values.

## 3. Results

### 3.1. Calibrated Density-Dependent Material Parameters

The optimization procedure determined the material constants appearing in Equations (4) and (5), which describe the density dependence of the elastic modulus and viscous coefficient of trabecular bone. The calibrated values are summarized in Table 2.

The identified parameters indicate that both elastic modulus and viscous resistance increase with increasing bone density. This trend suggests that denser trabecular bone provides greater load-bearing capacity together with stronger resistance to rate-dependent deformation.

According to the present analytical model, the cortical bone accounts for approximately 32% to 46% of the total vertebral load-bearing capacity, while the remaining mechanical resistance (54% to 68%) is supported by the trabecular core. This predicted global distribution is consistent with clinical imaging observations by Oppenheimer-Velez et al. [34], who reported a baseline cortical load-bearing share of approximately 29% in human lumbar vertebrae, confirming that both structural components play critical, coupled roles in overall vertebral strength.

### 3.2. Stiffness Prediction

The accuracy of the proposed model was evaluated by comparing the normalized stiffness values obtained from experimental force–displacement curves [18] with the effective modulus predicted by the model. Since the normalized stiffness is dimensionally equivalent to a modulus, this comparison enables a consistent evaluation of experimental and model results.

**Figure 3:**
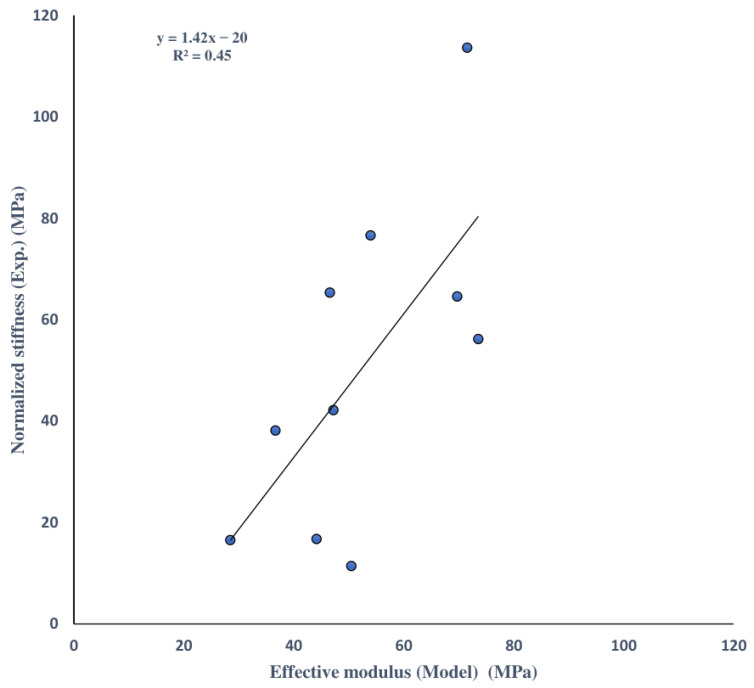
Correlation between predicted effective modulus and experimental normalized stiffness.

The effective modulus from the Kelvin–Voigt model includes both elastic and viscous effects. The model gives an R^2^ value of 0.45 for all samples (Figure 3), showing moderate agreement with the experimental results. In comparison, the purely linear elastic model gives a slightly lower R^2^ value of about 0.42.

Table 3 summarizes the effective modulus and fracture force values from the experiment and the model, together with the accompanying errors.

### 3.3. Fracture Force Prediction

**Figure 4:**
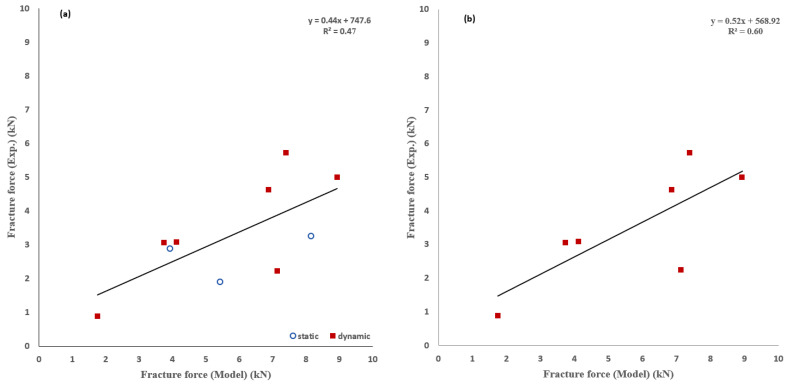
Predicted versus experimental fracture force for (**a**) all specimens, including static and dynamic tests, and (**b**) dynamically loaded specimens.

As shown in Figure 4, the model predicts fracture force and compares it with experimental data. Peak forces were obtained from compression tests reported in a previous study [18], with fracture force defined as the maximum recorded load.

Overall, the comparison between model predictions and experimental results yields a coefficient of determination of R^2^ = 0.47 (Figure 4a), indicating moderate agreement. In comparison, the purely linear elastic model yields a lower R^2^ value of 0.35, highlighting a more pronounced improvement when viscoelastic effects are included. When only dynamically tested specimens are considered, the correlation improves to R^2^ = 0.60 (Figure 4b). This improvement suggests that the viscoelastic formulation more effectively captures vertebral behavior under dynamic loading conditions.

### 3.4. Model Accuracy and Error Analysis

Prediction errors were examined to identify specimens with unusually large deviations between model predictions and experimental measurements. Grubbs’ test [35] identified specimen 5186L3 as a statistical outlier in both stiffness and fracture force prediction errors. A significance level of α = 0.05 was used. This specimen showed the largest errors in the dataset, with 341% error in effective modulus and 219% error in fracture force. After excluding this outlier, the coefficient of determination increased from R^2^ = 0.45 to R^2^ = 0.53 for effective modulus prediction and from R^2^ = 0.47 to R^2^ = 0.60 for fracture force prediction. For dynamically tested specimens, fracture force prediction improved more substantially, with R^2^ increasing from 0.60 to 0.88 after exclusion of the outlier specimen.

Specimen 5186T8, obtained from the same donor, also showed a relatively high effective modulus prediction error; however, it was not identified as a statistical outlier and was therefore retained in the primary analysis. These findings suggest that donor-specific factors and microstructural characteristics not captured by density alone may influence the mechanical response of the vertebrae.

**Figure 5:**
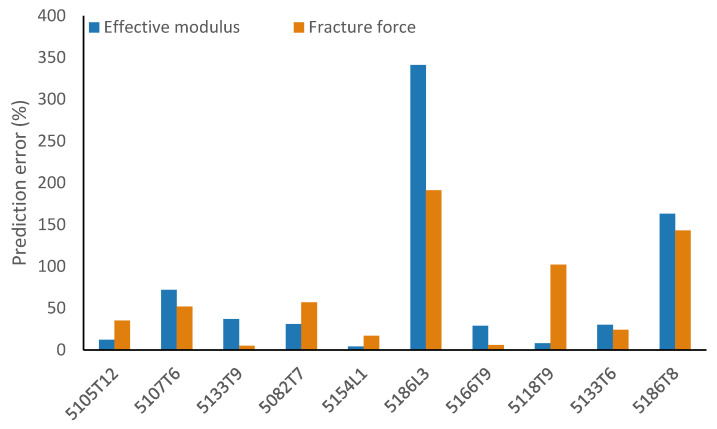
Comparison of effective modulus and fracture force prediction errors across all tested specimens. Values are presented in percentage (%).

It is worth noting that the predicted effective modulus and fracture force (Figure 5) for two specimens from the same donor (5186L3 and 5186T8) showed large positive errors. In both cases, the model clearly overestimated the results. This suggests that something other than density is affecting the mechanical response of these samples. Density is important, but it does not fully describe the trabecular structure. Other factors, such as anisotropy, connectivity, cortical thickness, possible microdamage, and specimen geometry, may also influence the dynamic behavior of the vertebra.

If only density is used, the mechanical properties may be overestimated, especially when the internal structure of the bone is degraded.

To better understand the source of these errors, the relationship between the error in elastic modulus and different density measures (cortical, trabecular, and apparent density) was studied.

Notably, specimens 5186L3 and 5186T8 exhibited significantly higher stiffness prediction errors compared to the rest of the cohort. This discrepancy is primarily driven by pronounced specimen-specific microstructural heterogeneity and localized architectural variations that cannot be fully captured by macro-scale density mapping alone. These cases represent extreme structural variances within the sample group. Despite these high-error cases, the overall framework maintains a robust capacity for tracking general mechanical trends across the broader specimen distribution.

It should be noted that both cortical and trabecular bone tissues are inherently heterogeneous, exhibiting spatial density distributions that influence localized stress concentrations. In the present analytical formulation, regional volumetric average densities were implemented as input parameters instead of continuous distributions. This simplification was intentionally adopted to preserve the mathematical tractability of the closed-form viscoelastic solver, as integrating spatial heterogeneity would yield extreme analytical complexity. Furthermore, from a clinical feasibility perspective, utilizing macro-level averaged parameters ensures that the model can function as a rapid screening tool independent of high-resolution voxel-by-voxel mapping workflows. This dual-compartment homogeneous approach represents a well-established paradigm in analytical spinal biomechanics, as demonstrated by Mizrahi et al. [36], Shirazi-Adl et al. [37], who successfully captured macro-structural vertebral responses using region-specific average properties.

## 4. Discussion

In this study, a density-dependent viscoelastic model was used to describe the mechanical behavior of human vertebral bone. The model relates the elastic response to bone density and also includes time-dependent behavior using a Kelvin–Voigt model. The predicted effective modulus values showed moderate agreement with the experimental data, which is reasonable because trabecular bone has high natural variability.

To support the reliability of the calibrated framework, the derived bone density ranges were benchmarked against established literature. The extracted trabecular density range (60.0 to 140.0 kg/m^3^) is consistent with the landmark database compiled by Morgan et al. [10] and Öhman-Mägi et al. [38], who reported human bone apparent densities in the range of 110 to 350 kg/m^3^ and 90 to 350 kg/m^3^, respectively, where vertebral trabecular regions naturally track the lower bounds of this spectrum. Similarly, the calculated cortical density range (553.1 to 709.9 kg/m^3^) aligns with the expected physiological bounds for human vertebral cortical shells, which exhibit lower continuum-level densities due to significantly higher localized porosity and thinning compared to long bones.

Regarding the prediction accuracy across the evaluated cohort, a notable localized inflation in stiffness error percentages was observed, with the maximum reported error (341%) attributable to a single specimen, 5186L3 (as presented in Table 3). To systematically evaluate these high-error cases, a formal Grubbs’ test for outliers (α = 0.05) was executed, which statistically identified specimen 5186L3 as a significant outlier within the dataset. A detailed post hoc assessment indicates that the elevated errors in specimens 5186L3 and 5186T8 are driven by a combination of a baseline systematic overestimation trend (scaling bias) and the inherent limitations of macro-density-based continuum mapping. Because the proposed analytical formulations rely primarily on macro-scale density inputs derived from QCT, the framework cannot fully account for severe patient-specific structural variations, localized anisotropy, or geometric asymmetries, which were highly pronounced in these specific configurations.

Crucially, when this statistical outlier is excluded purely for trend interpretation purposes, the predictive performance of the framework demonstrates strong fidelity, yielding an R^2^ = 0.53 for stiffness and an R^2^ = 0.88 for fracture force. For the remaining majority of the cohort (e.g., 5105T12, 5154L1, and 5118T9), the prediction errors fall within a highly acceptable and narrow envelope ranging from approximately 4% to 15%. Importantly, despite these observations, no specimens were selectively removed from the main analysis, ensuring a realistic and uncompromised representation of the framework’s screening performance under practical clinical conditions. These findings underscore that while the current simplified density-dependent approach provides a highly efficient, first-order baseline for rapid mechanical screening, incorporating microstructural architecture and localized anisotropy metrics remains a key objective for future expansions of the model to further mitigate systematic bias.

Samples with similar average density may still behave differently because their internal structures are not the same. Factors such as trabecular orientation, connectivity, and local density variations can strongly affect the mechanical response. Some variation in the data may also be related to the assumptions used in the model. In addition, the presence of an outlier specimen suggests that the model is sensitive to specimen-specific features that are not fully represented by density-based relationships. Although the Kelvin–Voigt model can represent rate-dependent behavior, it does not account for nonlinear behavior or damage. These effects may become more significant at higher loads and may explain some of the differences between the predicted and experimental results.

The relatively small improvement of the present model compared with the purely linear elastic model in stiffness prediction may be related to the limited effect of viscosity within the studied strain-rate range. Considering the natural variability of cadaveric bone and the assumptions used in the model, this level of agreement still indicates that the model can capture the general trend of stiffness across the specimens. In particular, the viscous coefficient increased with density, suggesting that denser trabecular bone may resist rate-dependent deformation more effectively. This observation is consistent with previous studies reporting that bone stiffness and strength increase with strain rate.

Additionally, in the present model, this behavior is represented through the viscous component, allowing the density-dependent viscous coefficient to reflect the combined effects of density and strain rate. A comparison with a purely linear elastic formulation showed that the viscoelastic model provided modest improvement in stiffness prediction and more pronounced improvement in fracture force prediction. Model performance also improved for specimens tested under dynamic loading conditions, indicating that the viscoelastic formulation became more important at higher strain rates.

For fracture force prediction, the model relies on an assumed failure strain. Although this is a common method, it introduces uncertainty because the actual failure strain may vary between specimens. Overall, the results suggest that density alone cannot fully explain variations in mechanical response. Microstructural characteristics and specimen-specific differences also appear to play an important role. Nevertheless, the model provides a useful representation of vertebral behavior under loading conditions related to spinal injury.

Compared with patient-specific finite element approaches, the present analytical framework has a lower computational cost, requires fewer CT-based inputs, and avoids complex meshing and solver procedures. These features may make the model more suitable for rapid evaluation and wider clinical use.

**Figure 6:**
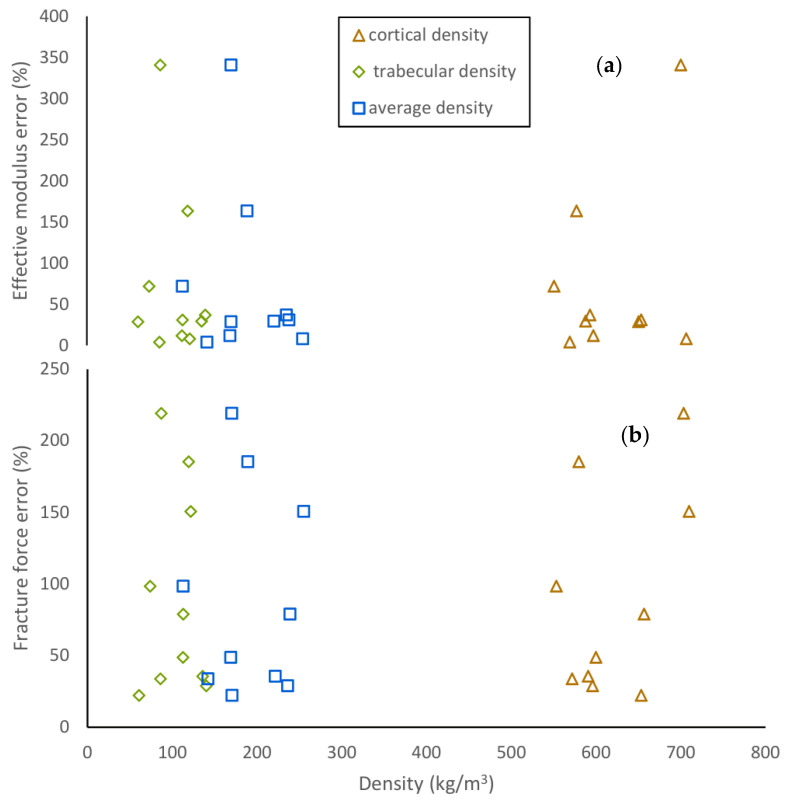
Prediction error in (**a**) effective modulus and (**b**) fracture force versus bone density for cortical, trabecular, and average density measures.

The dispersion observed between the model predictions and experimental measurements in Figure 6 highlights a classic paradigm in bone mechanics. Although bone ash density represents the premier predictor of vertebral structural capacity, it cannot single-handedly account for the entire spectrum of mechanical variability. Inherent donor-specific factors—including trabecular micro-architecture, localized microdamage, biological sex, age, and potential metabolic anomalies—exert pronounced secondary influences on structural failure. Due to the practical constraints of a sparse cadaveric dataset, incorporating these multifaceted variables into the constitutive relations was omitted to avoid mathematical overfitting.

Nevertheless, the fundamental significance of this study remains rooted in its capability to capture the overarching viscoelastic trend. By successfully embedding the primary density variable into a closed-form, strain-rate-dependent analytical framework, this model delivers instantaneous, physically meaningful biomechanical estimations, serving as an efficient alternative to high-computational-cost numerical models in preliminary screening scenarios.

A direct comparison between the parameter values obtained in this study and those reported in previous studies is not straightforward due to fundamental differences in the adopted modeling approaches. In many existing studies, the vertebral body is characterized by a spatially heterogeneous distribution of bone density, often incorporating a wide range of density values within a single vertebra.

In contrast, the present study simplifies this representation by assigning two effective density values to each vertebra, corresponding to cortical and trabecular bone. While this approach enables a more tractable and computationally efficient modeling framework, it inherently differs from methodologies that account for detailed density variations.

As a result, the parameters identified in this study reflect this simplified representation and cannot be directly compared with those derived from models based on fully heterogeneous density distributions. Nevertheless, the adopted approach captures the overall mechanical behavior of the vertebrae and provides a consistent framework for evaluating stiffness across specimens.

It should be noted that in an intact vertebral body, trabecular bone is enclosed by a cortical shell, which provides a confinement effect that can enhance its apparent compressive stiffness and strength. In the present study, trabecular behavior was primarily characterized based on uniaxial compression assumptions, and the confinement effect was not explicitly modeled.

Although the proposed effective modulus formulation incorporates the combined contribution of cortical and trabecular regions, it does not fully capture the local mechanical interactions arising from confinement. Therefore, the predicted mechanical response may differ from that of fully confined trabecular structures. This limitation should be considered when interpreting the results, and future studies may incorporate more detailed representations of trabecular–cortical interactions.

It is important to note that the implemented 10% compressive strain threshold represents a global macro-structural collapse criterion for the entire vertebral body, rather than a local tissue-level material yield. Biomechanically, the effective structural strain-at-failure in porous bone is non-linearly density-dependent; as described by empirical power-laws (e.g., the power-law function $\varepsilon_{y}=0.0081\rho^{-1.42}$), lower density regions undergo prolonged post-yield compaction where structural strains can dynamically escalate to 10% or higher. To rigorously evaluate the potential errors introduced by this single-threshold assumption, a parametric sensitivity analysis was executed across practical boundaries (7%, 10%, and 12%). At a 7% strain threshold, the model prematurely triggered structural failure, underestimating fracture forces by up to −14%. Conversely, a 12% threshold delayed structural collapse, overestimating load-bearing capacities by up to +283%. The absolute error magnitudes were globally minimized at the 10% threshold, proving it to be the mathematically optimized and balanced baseline for predicting macro-scale vertebral fracture force under multi-rate loading conditions.

Several limitations should be noted. First, the sample size is limited, which may contribute to the moderate agreement between the model predictions and the experimental data. Increasing the number of specimens could improve the results. Second, the vertebra was modeled as a cylinder, while real vertebrae have more complex and nonuniform geometries that can influence their mechanical behavior. Finally, the Kelvin–Voigt model does not account for nonlinear behavior or damage mechanisms, which may become important at higher load levels.

Furthermore, while the current analytical model utilizes a regularized geometric approximation, future investigations will focus on incorporating microstructural morphological indices extractable from QCT to better account for the irregular continuum geometry of the vertebral body.

Furthermore, the total sample size utilized in this study is limited to 10 human cadaveric specimens, which represents a recognized constraint of the current framework. Within this cohort, the sub-cohort sample size for quasi-static loading (three specimens) is small and disproportional relative to the dynamic fracture group (seven specimens). This limitation is primarily dictated by severe ethical restrictions, high acquisition costs, and the general scarcity associated with obtaining intact human cadaveric vertebral segments. While this cohort was sufficient for the initial development, proof-of-concept validation, and demonstrating the framework’s capability to capture multi-rate viscoelastic responses, the identified material constants should be considered as a foundational baseline. Expanding the specimen dataset in future investigations with larger, more balanced experimental cohorts will be essential to capture wider patient-specific biological variations and to further generalize and fine-tune these constitutive formulations across a broader demographic distribution.

While this investigation focuses strictly on axial compressive loading to establish a foundational analytical framework, the integration of multi-axial boundary conditions remains an objective for subsequent expansions of the developed formulations.

## 5. Conclusions

In this study, a density-dependent viscoelastic model was used to describe the mechanical behavior of human vertebral bone. The model combines a power-law relationship between elastic modulus and density with a density-dependent viscous term in a Kelvin–Voigt model. The results showed moderate agreement with the experimental data and improved prediction of mechanical response, especially under dynamic loading conditions. By including viscoelastic behavior, the model could represent the rate-dependent behavior of vertebral bone. The findings showed that although bone density is an important factor in the mechanical behavior of vertebral bone, it is not sufficient to fully explain the observed behavior. Other factors, such as bone microstructure, may also affect the behavior. Including these factors in the model may improve prediction accuracy. Overall, the model provides a useful approach for describing vertebral bone behavior under loading conditions related to spinal injury. The proposed model may also be a practical and computationally efficient alternative to more complex finite element models for preliminary assessment of vertebral strength.

## Figures and Tables

**Table 1:** Geometric characteristics, loading conditions, and density properties of the tested vertebral specimens.

Row	Bone ID	Loading Speed (mm/min)	Height (mm)	$\dot{\varepsilon }$ (s^−1^)	Cortical Area (mm^2^)	Trabecular Area (mm^2^)	Cortical Density (kg/m^3^)	Trabecular Density (kg/m^3^)
1	5105T12	12,000	24.0	8.33	169	1287	599.8	112.6
2	5107T6	12,000	13.8	14.49	50	565	553.3	73.7
3	5133T9	12,000	22.8	8.77	218	815	595.9	140.1
4	5082T7	12,000	19.2	10.42	282	933	656.6	113.1
5	5154L1	12,000	22.8	8.77	130	995	571.9	86.0
6	5186L3	12,000	15.6	12.82	192	1222	703.4	86.9
7	5166T9	12,000	19.8	10.10	149	654	653.3	60.7
8	5118T9	5	19.8	0.004	266	904	709.9	121.8
9	5133T6	5	19.8	0.004	137	589	590.7	136.0
10	5186T8	5	15.0	0.006	187	1041	579.8	119.3

**Table 2:** Calibrated material parameters.

Relationship	Parameter	Value	Unit
$E=a\rho^{b}$	a	25,834	$\mathrm{Pa}\cdot {\left(m^{3}/\mathrm{kg}\right)}^{b}$
	b	1.39	–
	c	107.8	$\mathrm{Pa}\cdot s\cdot m^{3}/\mathrm{kg}$
ƞ=cρ+d	d	−5938	Pa·s

Note: E = elastic modulus; ρ = density; ƞ = viscous coefficient.

**Table 3:** Comparison of experimental and model-predicted effective modulus and fracture force.

Row	Bone ID	Effective Modulus (Pa)			Fracture Force (N)		
		Experiment	Model	Error (%)	Experiment	Model	Error (%)
1	5105T12	47,179,898	42,180,495	12	6869	4623	49
2	5107T6	28,440,747	16,548,570	72	1749	881	98
3	5133T9	71,548,080	113,636,039	37	7391	5730	29
4	5082T7	73,553,063	56,176,909	31	8937	4997	79
5	5154L1	36,652,443	38,157,067	4	4123	3081	34
6	5186L3	50,453,129	11,444,487	341	7134	2234	219
7	5166T9	46,546,242	65,323,233	29	3738	3059	22
8	5118T9	69,706,787	64,562,385	8	8156	3253	151
9	5133T6	53,960,617	76,617,273	30	3918	2887	36
10	5186T8	44,134,980	16,762,866	163	5420	1899	185

## Data Availability

The raw data supporting the conclusions of this article will be made available by the authors on reasonable request.
